# Sterility in the Female, and Its Curability

**Published:** 1903-03

**Authors:** S. L. Kistler

**Affiliations:** Los Angeles, Cal.


For Texas Medical Journal.

### Sterility in the Female, and Its Curability.

BY S. L. KISTLER, M. D., LOS ANGELES, CAL.

The slight attention paid to this subject is possibly due to lack of information as to value of treatment, and partly because of disinclination for the burdens of maternity.

In this paper I shall avoid prolixity—as the subject is one which admits of great extension. Therefore, I shall of necessity treat the subject in a casual way only.

The conditions may be either acquired or congenital, and in taking the matter up I will consider the term "sterility," or "barrenness," to apply to women who, under favorable circumstances, fail to procreate.

I might be more exact, but to do so would only confuse, and my excuse for presenting this paper is to call attention to a subject of much importance to all practitioners, as it is fast becoming more and more widespread; and it behooves the doctor to use his best efforts to remedy the defect when called upon.

The causes of sterility may be enumerated as follows:

1. I believe that many cases of this condition, as well as of uterine disease in general, are the result of loss of sympathetic nerve force, the effect of disease, or injury arising from sexual demands.
2. An indefinite number of cases are undoubtedly the result of imperfect participation of the uterus in the sexual organs, and the consequent lessened respiratory action of the uterus.
3. Inactive ovaries.
4. Inactive condition of uterine mucosa.
5. Congenital deficiencies, anomalies of the hymen and malformation of the congenital tract.
6. Arrested development of ovaries and tubes.
7. Excessive acid reaction of secretion of vaginal mucus membrane and catarrhal changes in the mucous membrane of the uterus and tubes.
8. Vaginismus, neurasthenia, exhaustion, physical decadence, nymphomania.
9. Amenorrhea, dysmenorrhea, narrow internal and external os.
10. Flexions, versions.
11. Laceration of cervix, profuse leucorrhea, endometritis.
12. Gonorrhea, especially with involvement of adnexa.
13. Constitutional diseases and conditions.
14. Neoplasms, myomas, malignant growths, hypertrophy.
15. Ingestion of certain articles of diet and medicines.
16. Obesity.
17. Incompatibility.
18. Higher education.

TREATMENT.

When a case applies for treatment for sterility, we must bear in mind the fact that not all cases are imputable to women. Indeed, Dr. Vedder, who carefully examined 300 married women and their husbands who were childless, proved in effect that 70 per cent. of his particular cases were due to the man, either directly in consequence of functional impotence or absence of spermatozoa, or indirectly by infection of the woman by gonococci. In 30 per cent. of his cases the cause was due to the woman, and most generally owing to neoplasms or atrophy of the uterus.

Regarding the above quotation, I will say that I believe the reverse holds true in this part of the country.

Therefore, if treatment, either indicated or tentative, does not yield results within a reasonable time, we should proceed to investigate the husband's condition before we carry treatment further.

It behooves us to search diligently till we find the cause, and, if found incurable, the patient may be so informed, but I would advise all to go slow on this point, as our patient may, on the other hand, become enceinte, much to our chagrin and discomfiture. So, as before noted, search diligently for the cause. Get as complete a history of case as possible, for many factors may enter in, and heredity cuts a wide swath in helping us to arrive at a conclusion, and the poet who said, "Every man is an omnibus in which ride all his ancestors," may have spoken concerning the very case you have in hand.

Slight or minor diseases of the ovaries or tubes are said more often to cause the condition under consideration than more severe troubles. And this because of the intricate and difficult adjustment of the pavilion of tubes to ovaries, as this relationship may be set aside by the most trivial vice of structure or disease.

Arrest of development or disease of ovaries seldom cause sterility, as both are rarely diseased at the same time. Likewise many women conceive readily in whom orgasm is deficient or absent.

General treatment embraces remedies from borax down to Key-Tsi-Ching, a famous Japanese remedy, and on down to Lawsinia Inermis, a celebrated Arabian cure.

Probably our most beneficial remedies are belladonna, the auric salts and electricity, combined with such local treatment as is indicated.

Tone up nervous system and prohibit frequent coitus.

Tone up uterine mucosa, for conception may occur and result be blighted early if condition of mucosa is unfavorable.

For inactive ovaries and atrophy, we have valuable aids in electricity, protonuclein, ovarine, thyroids, sabal serrulata, ignatia and such other agents as are indicated and tend to develop and bring to maturity.

In congenital deficiencies, anomalies of hymen and malformation of tract, the help of the surgeon must be had, when he will remedy the condition, if possible.

Likewise we must call the surgeon in cases of laceration, neoplasms, and malignant growths, and, if thought necessary, in cases presenting myomas; however, it is supposed that they very seldom cause the condition.

Excessive acid reaction of vaginal secretion may be often corrected by use of suitable alkaline douches; further treatment will consist in toning up the mucus membrane.

Catarrhal conditions of not too severe type may be treated by applying solutions of arg. vit. or iodized phenol, and in cases presenting marked hurt in endometrium the above is profitably used before applying the curette.

Of course all malposition must be corrected as soon as possible. Graily Hewett reports thirty cases dependent upon flexions and versions cured by remedying the abnormality.

Vaginismus, neurasthenia, exhaustion, physical decadence, nymphomania, and all neuroses appearing demand careful investigation in order that source of trouble may be discovered, and if possible, remedied.

Amenorrhea, menorrhagia.

Since the days of Marion Sims we have been taught that the chief reason for sterility, attributable to the woman, is narrowness or flexions of the uterine canal. But when it is remembered that the narrowest pin-hole os will admit a sound on careful manipulation, which is many times larger than the self-propelling spermatozoid, it will be seen that this reasoning is scarcely satisfactory, and it is doubtless due to operative furor that the popularity of the stenosis and atresia theory of sterility continues, and since the time of Sims and Simpson, practically no form of treatment has been employed save some method for opening or enlarging the canal, a procedure which not only has been, as a rule, ineffective, but has been followed by morbidity and mortality, especially when slitting the cervix has been done.

Being satisfied with effect of electric currents and accepting the evidence offered, we believe that we have in it a treatment far and away in advance of any other means known at the present time. And Sims' assertion that sterility can only be cured by surgical interference is untenable;—rather use medication that raises the nutrition of the entire organism. And now we are told to feed or starve the woman according as we wish male or female progeny.

Constitutional diseases and conditions call for such measures as their respective types indicate.

In case of very large vagina, recourse may be had to artificial fecundation.

Avoid use of articles containing tannin, also forego use of antiseptics.

In case of absence of ovaries, "Knauer has reported successful pregnancy following transplantation of ovarian tissue."

Obesity produces sterility by mechanical pressure and by excess of fat in the blood and thus causes amenorrhea, and ripening and bursting of graaffian follicle is thus prevented. The treatment is obvious. Always bear in mind that such cases may be result of senile decay.

If gonorrhea has sealed up the ends of tubes, their restoration *ad integrum* is scarcely possible. However, as a *dernier ressort* an explorative incision will show if the condition be possibly remediable.

A noted American gynecologist has said, "I never knew a woman to bear a child after having had gonorrhea." This statement none of us believe.

Finally, in the treatment of sterility, we should bear in mind the fact that each case is a law unto itself, and we should study it as though it were the only case—remembering the great number of cases dependent upon slight causes.

### CONCLUSIONS.

1. The great majority of cases of sterility are dependent upon slight causes.
2. The greater number of cases are curable.
3. Many apparently hopeless cases are curable.
4. Length of time a case has persisted is no bar to treatment, providing organic change has not obtained that precludes possibility of cure.
5. Treatment used must always depend upon the case in hand.

